# Considerations on the Reciprocal Influence of the Physical Organization and Mental Manifestations

**Published:** 1856-05

**Authors:** A. O. Kellogg

**Affiliations:** Port Hope, Canada West


*Considerations on the reciprocal influence of the physical organization and mental manifestations.* By A. O. KELLOGG, M. D., Port Hope, Canada West.

*The Cerebral and Reproductive Systems — their Reciprocal and Sympathetic Influences.*—The intimate sympathy of these systems, both in health and when influenced by disease, furnishes an interesting subject for physiological, pathological and psychological investigation. In the field of purely physiological and pathological inquiry, the labor has been abundant, and our medical literature has been enriched with works of standard excellence. But when we contemplate the reproductive system, whether in health or disease, in its relations to the cerebral system and to psychology, we are forcibly struck with the paucity and imperfection of our knowledge. The recorded facts which bear upon the reciprocal influence of these systems, are widely scattered, and the attempt to collect, digest and systematize them, and make the necessary scientific deductions, would be a work of some importance, and one which would demand more time and space than is usually devoted to an article for a medical journal. I shall not, therefore, attempt even a synopsis of the physiology which is applicable to this subject, or enter into a labored and scientific investigation of the known sympathy which exists between these systems; but, presupposing a knowledge, on the part of the reader, of the physiology involved in the inquiry, confine myself to the pathological phenomena and their relations to psychology.

The influence of excessive and unnatural excitement of the sexual organs upon the mental faculties has long been recognized. In tables setting forth the supposed causes of insanity, this has long occupied a prominent place, and though its influence as a cause of insanity has no doubt been overrated, still it is undoubtedly great.* Of the probable causes of derangement in three hundred and sixty-six cases of insanity, occurring in both sexes—as recorded in the report of the Superintendent of the New York State Lunatic Asylum for the year 1852—eighty-seven, or nearly one-fourth of the whole number of cases, are reported to have arisen from causes directly connected with the reproductive system. In the report of the Superintendent of the same institution for the year 1853, of the supposed causes of insanity in four hundred and twenty-four cases, one hundred and seventeen, or more than one-fourth, appear to have had direct connection with some disturbance of the sexual organs. In the able and interesting report of Dr. Gray to the Managers of this Institution for 1853, of three hundred and ninety, seventy-two are supposed to have had some direct connection with derangement of these organs. From the above it would appear, that in nearly one-fourth of all the cases of insanity, occurring in both sexes, the disturbance of the generative organs was so marked as to be regarded as a *primary cause* of the mental derangement.

Whether the primary cause in these cases had its seat in the cerebral or the generative system, is a question no less interesting than difficult of solution, and one which could only be determined by an attentive consideration of the history of the cases reported, and a close observance

* See the testimony of the late Dr. Brigham in the Philips Will Case.—*Journal of Insanity*, vol. vi, p. 182.

of the true sequence of the symptoms in each. And, whether the primary link in the chain of morbid sympathies had its seat in the one system or the other, it is none the less important in relation to the reciprocal influence of the two.

As the functions of the reproductive system are far more important and intricate in the female than in the male economy, and as the pathological disturbances are, as a natural consequence of this, more frequent and interesting, we are, as a matter of course, to look to the female generative system for the most important illustrations of the sympathetic disturbances and reciprocal influences dependent upon these pathological lesions. Hence all the diseases which affect the female generative system have, at one time or another, been brought forward as causes of insanity. Even its most natural function, that of gestation, does, in some cases, by the peculiar change wrought in the female economy, and the train of inexplicable nervous symptoms which result, give rise to insanity; and there are cases on record of females who have been positively insane during the whole of each period of utero-gestation, but who recovered their mental health and strength soon after delivery. The cases in which some slight mental or moral disturbance during gestation has been observed are numerous; there are many on record, and every experienced practitioner is able, no doubt, to recall to mind such slight disturbance.

The influence which the menstrual function, even when performed apparently in a healthy manner, exercises upon the mental faculties and moral feelings of some females is exceedingly interesting to the intelligent and philosophical observer. In certain abnormal states of this function the influence is still more apparent. I have been told by intelligent females, accustomed to analyze their own feelings, that they felt far less mental energy during this state than in the intervals, and that they possessed far less control over the moral feelings than at other times, were more easily excited, and that the most trifling circumstances, which at other times would pass unheeded, have, in spite of every effort, greatly disturbed their equanimity. In some whom I have known, of a nervous, excitable temperament, their whole character appeared changed during the menstrual period, and from being cheerful, kind, firm, patient, and decided, they became morose, taciturn, wayward, fidgety, and impatient, frequently manifesting a certain nervous irritability bordering on hysteria, and were sometimes overcome by paroxysms of that interesting affection.

The changes which take place in the mental and moral faculties about the time of puberty, are in both sexes very interesting, particularly so in the female. These have been frequently alluded to by medical writers, as attendant upon a fuller development, and higher manifestations of vitality in the sexual organs. During these changes the nervous system exhibits increased susceptibility and sensibility, and not only the whole frame, but also the mental manifestations present greater activity of development. "The mind," says Dr. Copland, "acquires extended powers of emotion and passion, and the imagination becomes more lively. If, on the other hand, the uterine organs continue undeveloped, and the menstrual discharge does not appear, the mind is dull, weak, or depressed, and the emotions and passions are imperfect or altogether absent."*

---

* Dictionary of Medicine. Art. Menstruation, vol. ii, p. 959.

The young female, who, up to the time of these changes, has appeared, comparatively speaking, a non-sexual being, in her intercourse with her companions, playing in childlike innocence and unrestrained freedom with the opposite sex as with her own; ignorant and unconscious of the powers within her which are soon to be awakened from their slumbers, begins, as she now approaches the verge of womanhood, to be animated with feelings and desires to which she was before a complete stranger, and which she regards with a deep interest as the forerunner of something, she scarcely knows what, and which she feels inclined to cherish, yet shrinks from, as though she knew not whether they were of good or evil omen.

In her intercourse with her former playmates of the opposite sex, there appears gradually to have dawned upon her an interesting shyness and maidenly reserve. Expressions which before conveyed no meaning to her pure mind, and which even now are but partially understood, are yet sufficiently so to tinge her cheek, and cause her to shrink back instinctively, as from some foul and pestilential presence. Her likes and dislikes are stronger, and rendered more apparent to those around her. In short, the physical changes brought about in a limited period of time in the sexual system have wrought a complete change in the mental and moral character of the young girl, and this most interesting period of transition terminates in that of complete womanhood, with all its desires and its aspirations, its hopes and its fears, its joys and its sorrows.

But it is in connection with the various diseases incident to the female generative system that we are to look for the more curious illustrations of this cerebral sympathy. The abnormal mental state of many patients laboring under hysteria, menorrhagia, dysmenorrhea, amenorrhea and the affections intimately associated with uterine derangement, has long been observed by medical men. "In at least three cases out of four," says Dr. Francis, "I have found hysteria associated with uterine derangement, and the restoration of the menstrual function to its healthy state has proved the precursor of the removal of the hysterical annoyance." Hysteria, again, may manifest itself chiefly by disorder of the mental faculties, and the moral feelings and emotions. "The mental affections," observes Dr. Copland,* "connected with hysteria, may be referred, 1st, to certain states of monomania, among which excited desire, amounting in some cases to nymphomania, may be enumerated; 2d, to *ecstasis* and mental excitement, in some cases of a religious nature, in others of different descriptions; 3d, to a state of somnambulism; 4th, to a form of delirium, generally of a lively character, with which various hysterical symptoms are often conjoined: 5th, to various delusions, generally of a hypochondriacal kind, to which the patient may become subject, or even the victim, owing to the indulgence it may meet with from imprudently kind relatives; and, 6th, to a desire to feign various diseases, sometimes of an anomalous or singular form."

The subjoined remarks of this same acute and philosophical physician are so apposite that we cannot resist the temptation to transcribe them in this connection. "Hysterical females," says he, "are not merely capricious or whimsical, but they often become enthusiastic for a time in the pursuit of an object, or in cherishing an emotion by which they

---

* Dictionary of Medicine, vol. ii, p. 321.

have been excited. In many such cases the nervous excitement and vascular turgescence of the uterine organs determine the character of the mental disorder; elevating certain of the moral sentiments, or of the intellectual manifestations, to a state of extravagance, passing in some instances into delusiôn or monomania. Many cases of puerperal mania are merely extremes of the hysterical disorder of the moral and intellectual powers or states of the mind. All these more extreme forms of mental affection are observed only where, in connection with much local or uterine irritation, there is great deficiency of nervous energy generally, and of mental power in particular; or where, with such deficiency, there has been much injudicious culture, or perversion, or improper excitement of the imagination. Females sometimes become passionately attached to an object, and this passion may advance even to nymphomania or monomania. * * * * * * The hypochondriacal feelings, the desire to deceive, or to simulate various diseases, or the delusions which sometimes possess the minds of hysterical females may be classed with the foregoing, as requiring a similar plan of treatment. In all them the *intentions of cure* are, to remove irritation or vascular turgescence of the uterine organs; to improve the general health; to strengthen the nervous system; to calm the imagination, and to guide the moral impulses of the patient. The most efficient, however, of these means are not likely to be adopted by the patient. Few will resort daily to the shower bath, or even occasionally to terebinthinate enemata, or submit to a course of tonics, or to a suitable regimen, &c., while she belives her health but little affected. Even when the hysterical disorder is of a very painful kind, the variability or capricious state of her mind leads her to run from one physician to another before opportunity of administering aid is afforded to any. At last, the most notorious charlatans—particularly those who either *excite the body through the mind, or the mind through the body*, the animal magnetizers, the homœopathists, the St. John Longs of rubbing celebrity, and the Campbells of celestial-bed notoriety—fix her attention. At such medical bagnios there is something promising gratification as well as excitement, and at such places hysterical as well as hypochondriacal patients 'most do congregate.' "*

When we pass from the consideration of the influence of the reproductive system upon the cerebral, to take a view of the influence of the latter upon the former, we enter upon an enquiry possessing as profound an interest as any in the whole domain of science; and here again, as before, we have to look to the female economy for the most interesting facts and phenomena illustrative of this mysterious and inexplicable sympathy. The results of this influence, if we allow ourselves to believe the statements, and receive as evidence what is brought forward as fact in illustration of it, are indeed sometimes most extraordinary; and the unmistakable evidence of this which is from time to time presented to the medical observer, is sometimes so curious as to make him pause before rejecting, as the workings of a morbid imagination, the statements which are sometimes made by intelligent females whose veracity we cannot doubt, and whose motives for deceiving us we are unable to discover.

There are few physicians of experience who will not be able to call

* Dictionary of Medicine, vol. ii, p. 337.

to mind some extraordinary statements made to them by females in reference to this sympathy, which at the time, no doubt merely called forth a smile of incredulity or surprise at what was then regarded as the result of superstition, or of a morbid imagination, but which in after hours, has been seized upon by him "as food for reflection."

Take the following cases as examples in illustration of this. An intelligent lady once pointed out to me a large *maternal mark* on the body of her young daughter, which she accounted for in the following singular manner. At the time of her pregnancy she was once retiring to bed accompanied by a female friend who happened to be staying with her at the time. After having undressed herself, and as she was changing her underclothing, her companion observed a live toad hopping around the room, into which, by some means, it had found its way. Her companion caught up the creature, and threw it at her, hitting her on the bare body, causing her to shudder and experience the most intense feelings of disgust as the cold, slimy body of the toad came in contact with her person. When the child, at the time in her womb, was born, this mark on its body was found, the position of which, said the lady, corresponds precisely with the spot where her own was struck by the body of the toad. The result (supposing it to be such) of the mental impression in the following case is much more interesting.

Mrs. N., of T., a highly intelligent lady, of refined and cultivated tastes in art, particularly painting,--is the mother of several interesting daughters. Though highly intelligent children, with one exception they are not remarkable for personal beauty. The one which forms the exception possesses one of the most lovely and beautiful faces I ever beheld, the expression of which is so truly angellic as to attract the attention and call forth the admiration of every one who sees her. Once when conversing with the mother respecting her children, and remarking upon the extraordinary beauty of the one alluded to, she said to me that she had a peculiar theory of her own to account for the uncommon features of this child, so unlike any of the others or either of its parents. Her theory was this, and though strange, she considered it quite as sensible as some other theories brought forward to account for strange things.

While pregnant with this child, her husband, who is a man of wealth and a connoisseur in art, purchased a beautiful painting—a Madonna, either an original or some excellent copy of one of the old masters—and hung it in the drawing-room. She was enraptured with the sweetness, and beauty of this picture, and often sat for hours together with it hanging before her while at her work. To this circumstance Mrs. N. attributed the sweetness and beauty of this child's face, so unlike either of the others. Between the features and expression of this picture and those of the child there was certainly a similarity.

The manner of the life led by Letitia, mother of the great Napoleon, while pregnant with the embryo warrior, has been supposed to have influenced in a high degree his extraordinary organization, and helped to determine his character. She appears, by the concurrent testimony of all, to have been a woman of strong character, and was often placed in circumstances requiring its vigorous exercise; and at no period of her life do her resolution and firmness appear to have been more heavily taxed than during the civil war which desolated the island of Corsica, immediately before it became a part of France. Her husband was an officer under

General Paoli, and she followed him on horseback wherever he went, sharing his dangers; even when pregnant with Napoleon she appears not to have been deterred from this by the embarrassing circumstances of her delicate condition, but was exposed thereby to many perils which few women have nerve to endure, and even less to be able to carry a conception to its full time. There cannot be the slightest doubt that all this had a marked influence on the organization and character of the child which was then being evolved within her womb.

Cases in which death of the fœtus has been caused by some sudden shock, or some powerful mental or moral emotion in the mind of the mother are numerous. I have been told repeatedly by females who have given birth to dead children, that, after some powerful mental or moral emotion, they ceased directly to feel any movement of the fœtus, and have dated the death of the being within them from that moment. Of the extraordinary influence of mental emotion upon the secretion of milk, and through this upon the health of the nursing infant, we have many interesting examples; and it would be of the utmost practical importance to diffuse more information among nursing females on this subject I am confident that I have frequently seen the death of the nursing infant result from the ignorance of the mother of this important influence; and several cases are now presented to my recollection, where the life of the infant has been placed in the utmost jeopardy by its mother's nursing it when in a state of powerful mental excitement. Not long since I was called to see a child aged seven or eight months, which, up to a short time before my being sent for, had been in a most thriving condition, exceedingly healthy and robust. I found the child in a state approaching complete coma, in a condition much resembling that which results from hydrocephalus, or anæmia of the brain, as the result of some exhausting disease. It had suffered no such disease; and as the coma had come on suddenly, constipation of the bowels only having been observed as its forerunner, I felt puzzled to determine the true cause. After, however, a free action of the bowels, for which large doses of cathartic medicine were required, it rapidly regained its consciousness, and, after passing dark green stools for a number of days, completely recovered. The mystery which shrouded this case, and which I was not able to unravel at first, was soon, however, explained; for, in conversation with a near neighbor, I learned that the mother, who was a woman of very violent temper, had for a number of days been giving away to most intense paroxysms of rage, which had been expended upon her husband, for selling a piece of property against her wishes. During all this time she was nursing her child. I immediately requested the mother, if she wished to rear her offspring, of which she was passionately fond, to suspend nursing it under such a state of mental excitement; and if she could not control herself, and make up her mind to be quiet and cheerful, it would be advisable to wean the child, or employ a wet nurse, while giving the reins to her passion, and not allow its force to be expended upon the frail being who was innocently drawing its nourishment from her bosom. She appeared to feel the justice of the reproof, and was, doubtless, more careful for the future, as the child did well, though not weaned for several months after this occurrence.

Another lady, of a highly exciteable temperament, the mother of three children and who had frequently been under the medical care of the writer, gave birth to her first male child about one year since. The child was healthy, and appeared to thrive well for four or five weeks. Its mother, on first leaving her room, was, as is frequently the case with careful housewives, somewhat excited and vexed with the condition of things in the kitchen, and the "high life below stairs" which had evidently been led by the servants during her confinement. She was also excited, on the same day, by the arrival of some friends. In addition to this, after retiring to her room, she heard the child next in years to the infant, fall down a flight of stairs. She was much alarmed, had the child brought up to her room, screaming, with its nose bleeding and broken. She took it upon her lap, bathed its face, and after stanching the hemorrhage and quieting the child to sleep, she, most imprudently, and, though a highly intelligent person, ignorantly and innocently suffered the infant to nurse after this crowning excitement of the day. Its bowels became immediately deranged, the stools green, high fever and convulsions supervened, and the child died in great agony in less than three days, with all the symptoms of violent information of the bowels. Such cases ought to be a warning to all mothers, and the conscientious physician will be doing his duty in advising such excitable persons of these dangers, that they may avoid the pain consequent upon the sickness and frequently the death of their offspring, of which they ave been, though ignorantly, and perhaps innocently, the cause.

"No secretion," says Dr. Carpenter,* "so evidently exhibits the influence of the depressing emotions as that of the mammæ." Sir Astley Cooper, in his able Treatise on the Breast, says, "The secretion of milk proceeds best in a tranquil state of the mind; then the milk is regularly abundant, and agrees well with the child. On the contrary, a fretful temper lessens the quantity of milk, makes it thin and serous, and causes it to disturb the child's bowels, producing intestinal fever and much griping. *Fits of anger* produce a very irritating milk, followed by griping in the infant, with green stools. Grief has a great influence on lactation, and consequently upon the child. The loss of a near and dear relative, or a change of fortune, will often so diminish the quantity of milk as to render adventitious aid necessary for the support of the child. Anxiety of the mind has also the same effect. The reception of a letter which leaves the mind in anxious suspense lessens the draught, and the breast becomes empty. If the child be ill, the mother is anxious about it; she complains to the medical attendant that she has little milk, and that her infant is griped, and has frequently, frothy, and green motions. *Fear* has the same influence: the apprehension of the brutal conduct of a drunken husband will, for a time, stop the secre ion. Terror, which is sudden, and great fear instantly stop the secretion."

From the great importance which attaches itself to this subject, I deem no excuse necessary for transcribing the following cases, the most interesting on record in reference to this matter.

A ca penter fell into a quarrel with a soldier billeted in his house, and was set upon by the latter with his drawn sword. The wife of the carpenter at first trembled with fear and terror, and then suddenly threw herself furiously between the combatants, wrested the sword from the soldier's hand, broke it in pieces, and threw it away. During the tumult some neighbors came in an separated the men.

---

*Human Physiology, p. 475.

While in this state of strong excitement, the mother took up the child from the cradle, where it lay playing, and in the most perfect health, never having had a moment's illness; she gave it the breast, and in so doing sealed its fate. In a few minutes the child left off nursing, became restless, panted, and sank dead upon its mother's bosom. The physician, who was called in instantly found the child lying in the cradle as if asleep, and with its features undisturbed; but all his resources were fruitless: it was irrecoverably gone. In this interesting case the milk must have undergone a change which gave it a powerful sedative action upon the susceptible nervous system of the child.

A lady, having several children, of which none had manifested a tendency to cerebral disease, and of which the youngest was a healthy infant a few months old, heard of the death, from acute hydrocephalus, of the infant of a friend residing at a distance. The circumstance made a strong impression upon her mind, and she continued to dwell upon it. One morning, shortly after having nursed it, she laid the infant in the cradle, asleep, and apparently in perfect health; her attention was shortly attracted to it by a noise, and, on going to the cradle, she found her infant in a convulsion, which lasted for a few minutes, and then left it dead. Now, although the influence of the mental emotion is less unequivocally displayed in this case than in the last, it can scarcely be a matter of doubt, since it is natural that no feeling should be stronger in the mother's mind, under such circumstances, than the fear that her own beloved child should be taken from her, as that of her friend had been; and it is probable that she had been particularly dwelling upon it at the time of nursing the infant on that morning.

A mother had lost several children, in early infancy, from convulsive disorder. One infant, however, survived the usually fatal period; but whilst nursing him one morning, she had been strongly dwelling on the fear of losing him also, although he appeared a very healthy child. In a few minutes after the infant had been transferred to the arms of the nurse, and while she was urging her mistress to take a more cheerful view, directing her attention to his thriving appearance, he was seized with a convulsive fit, and died almost instantly."*

Mr. Wardrop, in the *Lancet*, No. 516, states, that having removed a tumor from behind the ear of a mother, all went on well, until she fell into a violent passion, and the child being suckled soon afterwards, died in convulsions. He was sent for hastily to see another child in convulsions, after taking the breast of a nurse who had just been severely reprimanded, and was informed by Sir R. Croft that he had seen many similar instances.

Three cases are recorded by Burdach (Physiologie, § 522,) in one of which the infant was seized with convulsions on the right side, and hemiplegia on the left, on nursing after the mother had met with some distressing occurrence.

Another case was that of a puppy, which was seized with epilepsy, on sucking its mother after a fit of rage.

The influence of strong mental emotion upon the menstrual secretion is very marked. There are few women of intelligence who have not noticed this fact, and this influence is particularly marked in any of the

* Op. cit., 475-6.

usual disorders of menstruation. Menorrhagia is almost invariably aggravated by powerful mental emotions. Som eforms of dysmenorrhea are not only caused, but rendered more painful by mental or moral disturbance. Acute suppression of the menses may arise, says Churchill, from a bodily or *mental* shock received either just previous to, or during menstruation; and gives, in a note, the following inte esting illustration of this. Almost all the women, says he, who are sent up to Richmond Penitentiary, after being tried at the Recorder's Court, labor under suppression of the menses, in consequence of the mental agitation and distress they have undergone. But it is unnecessary to multiply illustrations of this, as I am anxious not to be led by the interest which attaches itself to this subject to exceed the limits within which I proposed, at the commencement, to confine myself.—*American Journal of Insanity.*

